# Amino acid positions near the active site determine the reduced activity of human ACOD1 compared to murine ACOD1

**DOI:** 10.1038/s41598-023-37373-w

**Published:** 2023-06-26

**Authors:** Fangfang Chen, Israfil Yalcin, Mingming Zhao, Chutao Chen, Wulf Blankenfeldt, Frank Pessler, Konrad Büssow

**Affiliations:** 1 Department of Structure and Function of Proteins, Helmholtz Centre for Infection Research, Braunschweig, Germany; 2 Research Group Biomarkers for Infectious Diseases, TWINCORE Centre for Experimental and Clinical Infection Research, a Joint Venture Between Hannover Medical School and the Helmholtz Centre for Infection Research, Hannover, Germany; 3 Institute for Biochemistry, Biotechnology and Bioinformatics, Technische Universität Braunschweig, 38106 Braunschweig, Germany; 4 Centre for Individualised Infection Medicine, Hannover, Germany

**Keywords:** Enzyme mechanisms, Enzymes, Molecular evolution, Evolutionary genetics, Dendritic cells, Monocytes and macrophages, Antimicrobial responses, Innate immunity

## Abstract

*cis*-Aconitate decarboxylase (ACOD1, IRG1) converts *cis*-aconitate to the immunomodulatory and antibacterial metabolite itaconate. Although the active site residues of human and mouse ACOD1 are identical, the mouse enzyme is about fivefold more active. Aiming to identify the cause of this difference, we mutated positions near the active site in human ACOD1 to the corresponding residues of mouse ACOD1 and measured resulting activities in vitro and in transfected cells. Interestingly, *Homo* *sapiens* is the only species with methionine instead of isoleucine at residue 154 and introduction of isoleucine at this position increased the activity of human ACOD1 1.5-fold in transfected cells and 3.5-fold in vitro. Enzyme activity of gorilla ACOD1, which is almost identical to the human enzyme but has isoleucine at residue 154, was similar to the mouse enzyme in vitro. Met154 in human ACOD1 forms a sulfur-π bond to Phe381, which is positioned to impede access of the substrate to the active site. It appears that the ACOD1 sequence has changed at position 154 during human evolution, resulting in a pronounced decrease in activity. This change might have offered a selective advantage in diseases such as cancer.

## Introduction

*cis*-Aconitate decarboxylase (ACOD1) catalyses the decarboxylation of *cis*-aconitate to produce itaconic acid, which is a key immunomodulatory metabolite of activated macrophages. It regulates cytokine production and inhibits viral replication^1,2^ and also plays roles in immune defences against bacterial pathogens^3^.

Published data suggest that mouse macrophages produce higher levels of itaconic acid than human cells, even though no direct comparison has been reported. Concentrations of 1.5–8 mM have been found in the mouse macrophage cell line RAW264.7 and in primary mouse bone-marrow-derived macrophages (BMDM) upon activation with LPS^4–6^. Levels in the human macrophage-like cell line dTHP1 were low upon LPS activation and simultaneous treatment with LPS and IFNγ was required to reach an intracellular itaconate concentration of 460 µM^7^. Primary human macrophages contained only approx. 60 µM itaconate upon LPS activation^5^.

**Figure 1:**
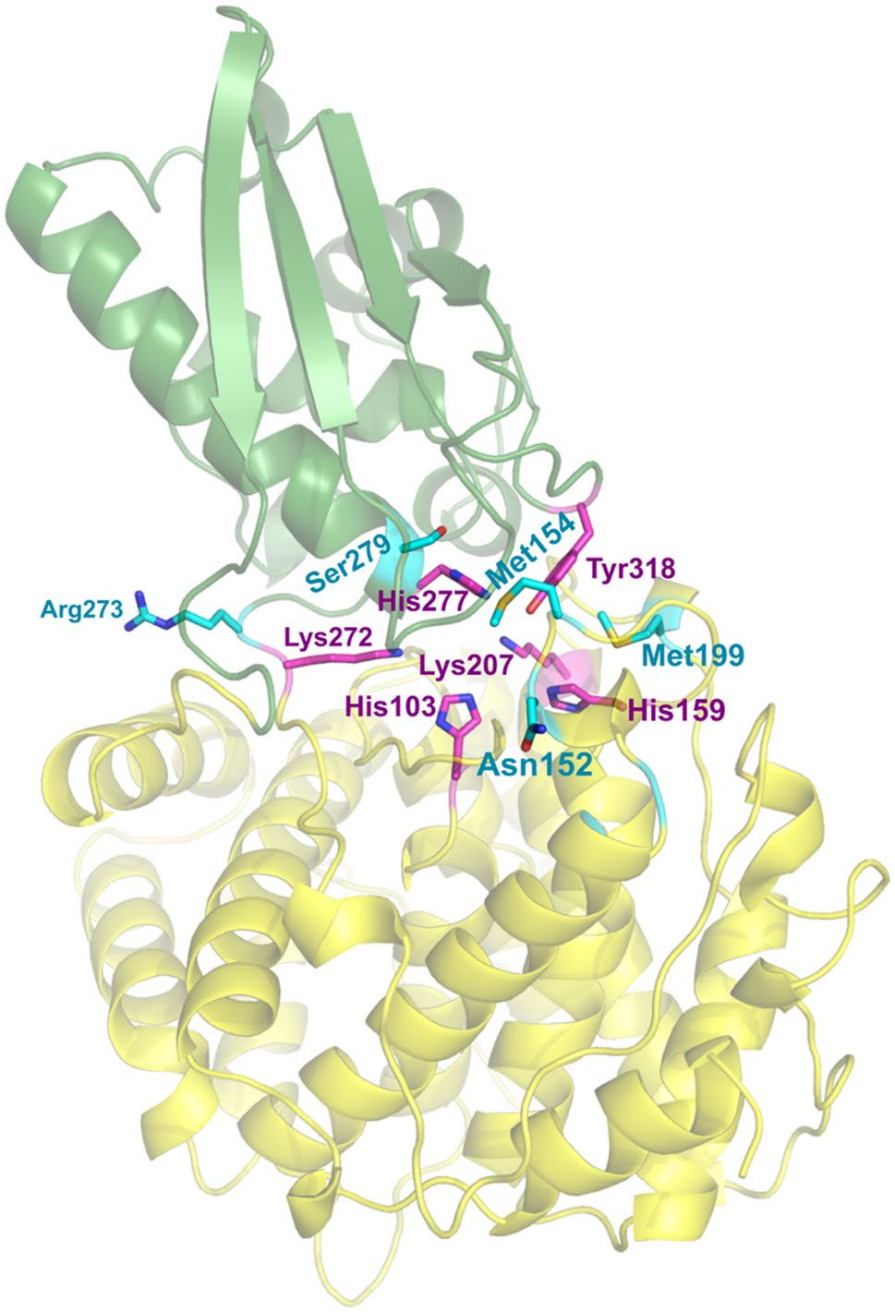
Crystal structure of a subunit of the human ACOD1 homodimer (PDB ID code 6R6U)^8^. Mutated residues differing between human and mouse ACOD1 are shown as sticks in cyan and conserved active site residues as magenta sticks. All these residues are close to the interface between the smaller (green) and larger (yellow) domain.

Correspondingly, the mouse ACOD1 enzyme is more active with a significantly higher catalytic rate constant *k*_cat_ of 4.9 s^−1^ in comparison to the human enzyme with 0.9 s^−1^^8^. The Michaelis-constants *K*_*M*_ were similar. The active site residues of both enzymes are completely conserved^8^, suggesting that the higher activity of the murine orthologue is determined by residues outside the active site. The mechanism of ACOD1 catalysis includes an opening of the active site by tilting the smaller domain relative to the larger domain (Fig. 1)^8,9^. This opening is required for the substrate to get to the active site. It is expected that the enzyme’s activity is determined not only by the conserved active site residues interacting with the substrate, but also by residues involved in the opening and closing movements. To determine the structural basis for the difference in ACOD1 activity between mice and human, we investigated residues near the active site and at the domain interface that might be relevant for the higher activity of mouse ACOD1 (mACOD1)^8,9^.

## Results

**Figure 2:**
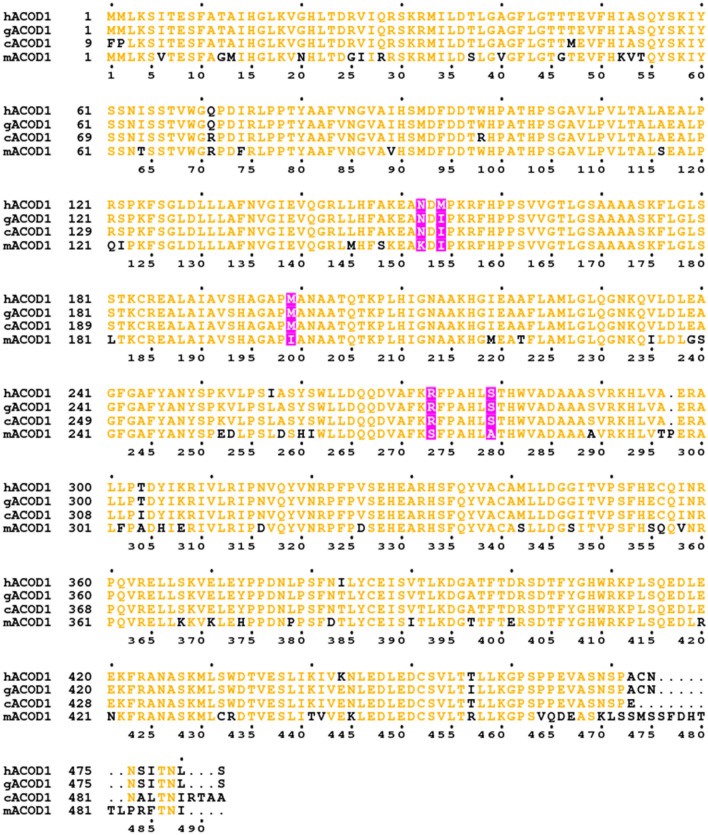
Sequence alignment of ACOD1 from human (IRG1_HUMAN), gorilla (G3RFY3_GORGO), chimpanzee (H2Q7N6_PANTR) and mouse (IRG1_MOUSE). Positions that were mutated are displayed with a pink background and non-conserved residues are shown in black.

**Figure 3:**
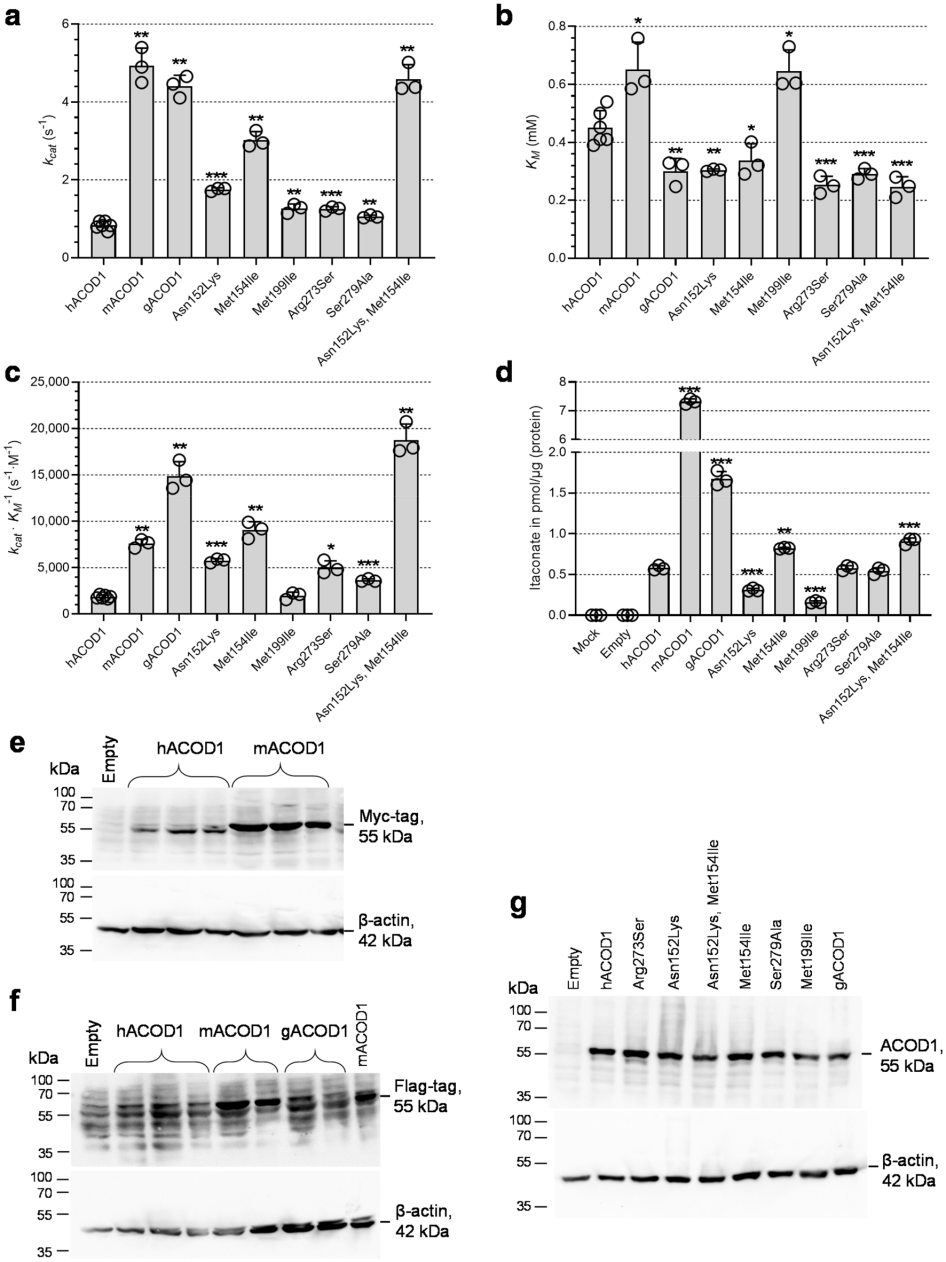
Activities of ACOD1 mutants. (**a–c**) *k*_*cat*_, *K*_*M*_ and *k*_*cat*_/*K*_*M*_ values of wild-type human, mouse and gorilla ACOD1 and human ACOD1 mutants, resulting from in vitro enzyme assays using purified enzymes and HPLC/UV quantification of itaconate production. (**d**) Proteins corresponding to (**a**) were expressed in transfected A549 cells and intracellular itaconic acid was measured by LC/MS 24 h post transfection. Individual measurements are represented by open circles and error bars represent standard deviations. Significant P-values calculated with Welch's unequal variances t-test for comparison with hACOD1 values are indicated by asterisks: *P < 0.05, **P < 0.01, ***P < 0.001 (Supplementary Table 1). (**e–g**) Immunoblots of ACOD1 proteins overexpressed in transfected A549 cells. (**e**) Overexpressed hACOD1 and mACOD1 plasmids were detected by Myc-tag antibody (three independent transfections). (**f**) Overexpressed hACOD1, mACOD1 and gACOD1 detected by Flag-tag antibody. (**g**) Overexpressed wild-type and mutant hACOD1 and gACOD1 detected by anti-human ACOD1 antibody. Loading internal control is β-actin. Empty, transfection with the empty pCMV6-Entry vector. Original blots are presented in Supplementary Figs. 1–3.

We selected five amino acid positions close to the active site and to the interface of the protein’s two domains, which differ between human and mouse ACOD1 (Fig. 1). We individually mutated the corresponding amino acids of the human sequence to their mouse counterparts. The mutations were Asn152Lys, Met154Ile, Met199Ile, Arg273Ser and Ser279Ala (Fig. 2). Recombinant proteins were produced in *E.* *coli* and their activity was tested in vitro (Fig. 3a–c). In parallel, A549 cells (which do not express the endogenous *ACOD1* gene) were transfected with wild-type and mutated plasmids, followed by measurement of intracellular itaconic acid concentrations (Fig. 3d). All mutations had a positive effect on the catalytic rate constant *k*_cat_ in vitro (Fig. 3a). Especially for the Met154Ile mutant, *k*_cat_ and itaconic acid production in cells increased strongly in comparison to wild-type human ACOD1 (hACOD1). The mutations also slightly improved substrate affinity and resulted in decreased Michaelis-constants *K*_M_ for all mutants except Met199Ile (Fig. 3b). A double mutant combining the mutations with the highest effect on *k*_cat_, Asn152Lys and Met154Ile, had an even higher *k*_cat_ value. Notably, this double mutant had the highest catalytic efficiency (*k*_cat_/*K*_M_ ratio) of all tested enzymes (Fig. 3c). The results of the in vitro assay and the transfection experiments corresponded well. However, while the Asn152Lys and Met154Ile double mutant had a similar activity as mouse ACOD1 in vitro, the transfection experiments with mouse ACOD1 resulted in much greater itaconic acid accumulation as compared to transfection with the double mutant of hACOD1 (Fig. 3d). This discrepancy can be explained by higher expression of mACOD1, in comparison to the human enzyme and the mutants. In order to measure expression levels of the expressed proteins, we made use of their C-terminal Flag and Myc tags. The mouse protein was readily detected in immunoblots with antibodies either against the Myc-tag or Flag-tag of the recombinant proteins, and the signal was considerably stronger in comparison to human or gorilla ACOD1 (Fig. 3e,f). Human wild type and mutant ACOD1 and gorilla ACOD1 (gACOD1) were all expressed at a similar level (Fig. 3g). It, therefore, appears that mACOD1 is expressed more efficiently than the human or gorilla sequences in our transfection system.

Interestingly, the methionine at position 154 is specific to humans, and sequences of Neandertals and Denisovans that we analysed also have the Met154 codon. Essentially all other known mammalian ACOD1 sequences, including those of other hominids, have an isoleucine at position 154 (Fig. 2). This indicates that Met154 was specifically acquired during human evolution before the population split between humans, Neandertals and Denisovans. Because Met154 is human-specific, hACOD1 was expected to have a lower activity than ACOD1 from our closest relatives, chimpanzee and gorilla. Gorilla and human ACOD1 differ at 5 positions (Fig. 2). Recombinant gorilla ACOD1 was highly active both in the in vitro assay and in the transfected cells and was more active than the Met154Ile mutant of hACOD1 (Fig. 3a,d). Chimpanzee ACOD1 (cACOD1) could not be produced in our *E.* *coli* system and it was expressed only weakly in the transfected cells, without detectable itaconic acid production.

**Figure 4:**
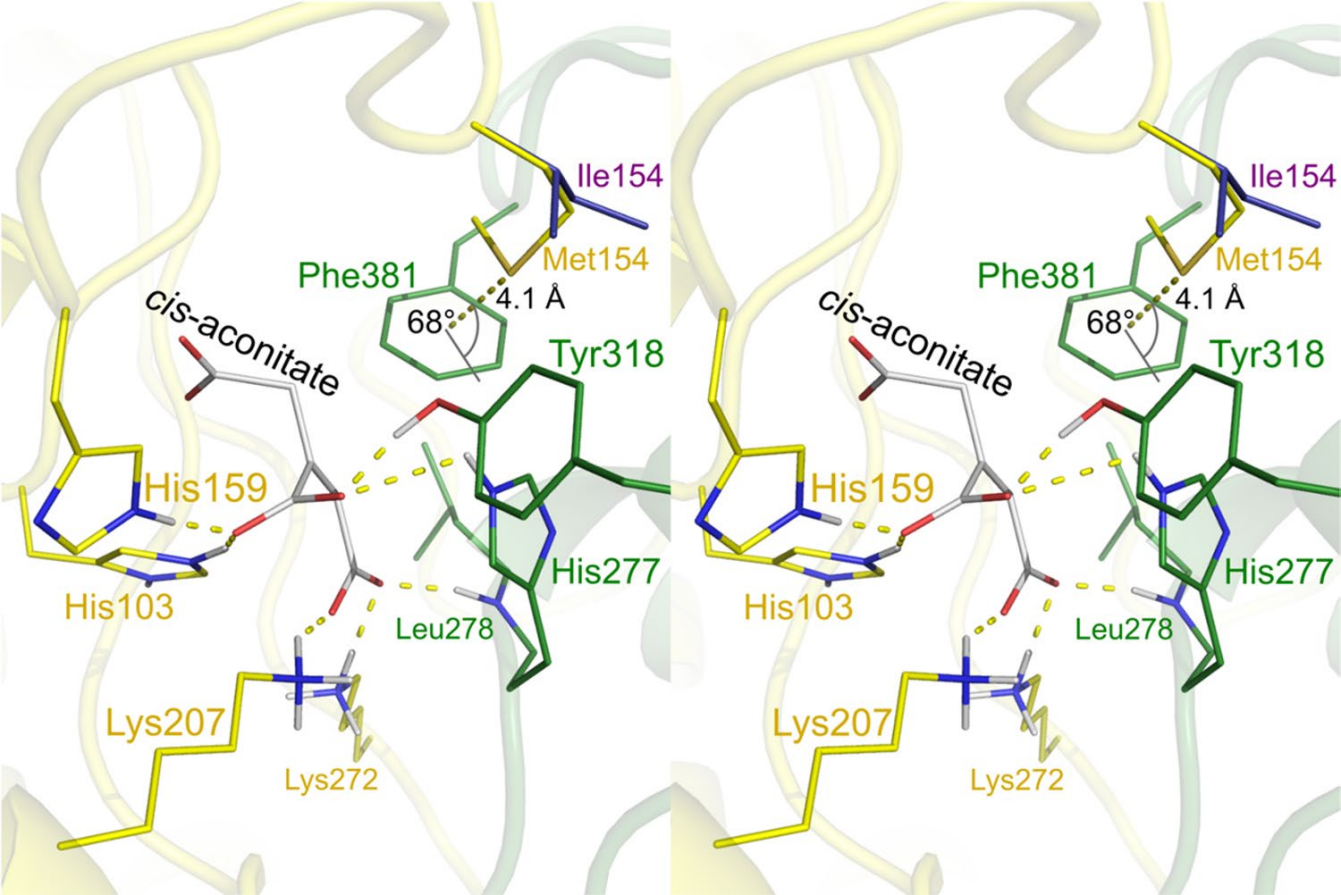
Stereo view of the hACOD1 active site and the sulfur-π bond between Met154 and Phe381. The active site of the hACOD1 structure (PDB ID code 6R6U) is shown with the putative conformation of the substrate *cis*-aconitate described in Chen et al.^8^. In this model, the substrate is bound by residues of the larger domain (yellow) and the smaller domain (green). The sulfur atom of Met154 is located at 4.1 Å distance from the Phe381 ring centre (dashed yellow line) at an angle of 68° to the plane of the aromatic ring. The Ile154 residue of the aligned mACOD1 structure of Chun et al.^9^ (PDB ID code 7BR9) is shown in purple.

A sulfur-π (Met-aromatic) bond^10^ between Met154 and Phe381 was identified by the computer program MetAromatic^11,12^ (Fig. 4, Supplementary Table 2). This human-specific interaction connects the larger and the smaller domain, suggesting that it stabilizes the enzyme’s closed conformation.

## Discussion

A cause for the different activities of mouse and human ACOD1 was identified. It was found that a human-specific residue, Met154, markedly reduces the activity of ACOD1. All other hominid ACOD1 sequences have an isoleucine at this position. The gorilla enzyme was found to be even more active than the mouse enzyme. This was unexpected, as the sequences of gorilla and human ACOD1 are 99% identical (Fig. 2). It suggests that during the evolution of *Homo* *sapiens* from the last common primate ancestor, a mutation at position 154 to methionine was acquired, leading to several-fold lower activity of the enzyme. Thus, it is expected that itaconate levels are lower in human ACOD1-expressing cells in comparison to other hominids.

Changing Asn152 to Lys also increased activity in the present study, especially in the double mutant with Met154Ile. We have previously identified a variant allele of human *ACOD1* in African ethnicity that changes Asn152 to Ser, leading to a 50% increase in enzyme activity^8^, underlining the importance of this position for enzyme activity. Asn152 is conserved in primates, but is not well conserved throughout mammals.

Human evolution from a common primate ancestor was driven by large changes in genome structure, by gene duplications and changes in non-coding sequences that regulate gene activity^13^. In addition, non-synonymous point-mutations in protein-encoding sequences resulted in gene inactivation. Only a small number of human-specific amino acid substitutions were identified that resulted in changes in protein function and biochemical properties. The brain-related proteins FOXP2^14,15^, MCPH1^16^ and ASPM^17^ and the male reproduction-associated protamines PRM1 and PRM2^18^ contain amino acid substitutions that arguably were targets of positive selection, indicating that the substitutions have an effect on protein function^19^. The Met154 in hACOD1 represents a single amino acid change during human evolution for which the biochemical consequences could be clarified.

The mechanism of ACOD1 catalysis includes an opening of the active site by tilting the smaller domain relative to the larger domain (Fig. 1)^8,9^. The side chains of residues 152 and 154 are located at the interface of the smaller and larger domain, and residue 154 also lines a hydrophobic pocket in the active centre. A sulfur-π bond was identified between Met154 and Phe381 of hACOD1, which links the two domains and stabilizes the enzyme’s closed conformation (Fig. 4). Sulfur-π bonds yield significant additional stabilization in comparison to purely hydrophobic interactions^20,21^. A higher stability of the closed conformation of hACOD1 would reduce substrate access to the active site and could thereby decrease overall enzyme activity.

The catalytic rate constant *k*_*cat*_ of mACOD1 was 5.9 fold higher than of hACOD1 in the present study. The catalytic efficiency *k*_cat_/*K*_M_, which is a measure of enzyme catalysis at low substrate concentrations, differed by a factor of 4.1. It should be noted that these in vitro enzyme parameters are not the only predictors of itaconate levels upon macrophage activation. Rates of transcription and translation and transcript as well as protein stability are also important, as are the rate of itaconate catabolism, secretion into the extracellular environment, and substrate availability. The higher activity of mouse ACOD1 may therefore not be the only reason for the much higher levels of itaconate in mouse cells. Further research is needed in order to identify the relative contributions of all parameters that ultimately determine intracellular itaconate accumulation.

Itaconate is an immune-regulatory compound with beneficial or detrimental effects in different kinds of diseases. It is an anti-infective and anti-inflammatory immunometabolite^22^. On the other hand, ACOD1 has also been reported to enhance tumour growth and reduce T-cell activity^23–26^ and aggravate sepsis in a mouse model^27^. One may hypothesize that the change to Met154 in human ACOD1, reducing itaconic acid synthesis, could contribute to a higher resistance against cancer, whereas the frequent Asn152Lys variant counteracts this reduction of enzyme activity, potentially improving host defence against infections. However, it is difficult to infer from the above reports about the role, if any, of ACOD1 activity in human evolution because most functional studies on ACOD1 (and itaconate) have been conducted in mouse models. Nonetheless, the differences between murine and human ACOD1 reported herein can serve as stepping stones for further studies on human ACOD1 function.

## Materials and methods

### Plasmids

Plasmids are listed in Supplementary Table 3. Plasmids pCAD29 and pCAD39 were used for *E.* *coli* expression of residues 4–461 of hACOD1 (GenBank NM_001258406) and residues 4–462 of mACOD1 (GenBank NM_008392) with N-terminal StrepTagII and TEV protease cleavage site^8^. Transfections were done with plasmids pCMV6Entry-hIrg1 and pCMV6Entry-mIrg1 for expression of full-length hACOD1 and mACOD1 with C-terminal Myc-tag and Flag-tag. Single point mutations were introduced into the hACOD1 expression vectors pCAD29 and pCMV6Entry-hIrg1^8^ by QuikChange mutagenesis (see Supplementary Table 4 for QuikChange primers).

The cACOD1 and gACOD1 plasmids for expression in *E.* *coli* were cloned with the Golden Mutagenesis method, using pCAD29_hIRG1_4-461_pvp008 as the template^28^. Primers were designed with the GoldenMutagenesis R library (see Supplementary Tables 5 and 6 for primers and PCRs). All primers had the tail GCGGGTACCGGTCTC, including a KpnI site. PCR products were cloned into the KpnI site of the vector pUC19exBsaI. pUC19exBsaI, a gift of Martin Bommer (Max Delbrück Center, Berlin, Germany), was derived from pUC19 by removing its BsaI site by introducing a silent point mutation. The resulting clones were used in Golden Gate reactions^29^ with the backbone vector pET-T7pro-ter, also a gift of Martin Bommer. pET-T7pro-ter was derived from pET-28a(+) by replacing the sequence from the NcoI site to the NotI site (CCATGG…GCGGCCGC) with the sequence CAATGAGAGACCGGT ACCGGTCTCAAGGTT AGTAAGCGGCCGC for Golden Gate cloning with BsaI (underlined). For Golden Gate cloning, the pET-T7pro-ter vector was diluted to 50 ng/µl (14.6 pM). The pU19exBsaI-derived plasmids were diluted to twice the molar concentration of pET-T7pro-ter (29.2 pM). The reactions were set up on ice with 1 µl of each plasmid, 2 µl T4 ligase buffer (NEB), 1 µl BsaI-Hfv2 (NEB) and 0.5 µl T4 DNA ligase (400 U/µl, NEB) and water to 10 µl and were incubated for 30 cycles of 5 min at 37 °C and 5 min at 16 °C, followed by incubation at 16 °C overnight. The reactions were heated for 5 min at 60 °C and then 5 µl were used for transformation of 50 µl OneShot OmniMAX 2 T1 chemically competent cells. This resulted in the clones pCAD161 (chimpanzee ACOD1, cACOD1) and pCAD183 (gACOD1). cACOD1 and gACOD1 cDNA sequences were cloned into pCMV6Entry by SLIC cloning^30^. The vector backbone fragment was generated by EcoRI and NotI digestion of pCMV6Entry-hIrg1. The full open reading frames of cACOD1 and gACOD1, including the missing terminal codons, were obtained by PCR from *E.* *coli* expression plasmids with long primers (Supplementary Tables 5, 6). For cACOD1, a full-length ORF (cACOD1.1, pCAD181, primers cCAD-pCMV6-LF and cCAD_1-480_pCMV6_R) and an N-terminally truncated ORF, starting at position 11 (cACOD1.11, pCAD180, primers cCAD-pCMV6-SF and cCAD_1-480_pCMV6_R), were cloned with the Zero blunt TOPO cloning kit (Thermo Fisher Scientific), generating clones TOPO4 and TOPO5. The N-terminus of the truncated cACOD1.11 ORF is the same as in hACOD1 (MMLKSITES…). The inserts of TOPO4 and 5 clones were SLIC cloned into the pCMV6Entry vector by using a QuickFusion kit (Absource Diagnostics), generating clones cACOD1.11 (pCAD180) and cACOD1.1 (pCAD181). gACOD1 was SLIC cloned into pCMV6Entry directly.

### Enzyme assays

ACOD1 proteins were produced in *E.coli* and purified as described^8^, using plasmids pCAD29 and pCAD39 for wild-type hACOD1 (aa 4–461) and mACOD1 (aa 4–461). A detailed protocol is available at protocols.io (10.17504/protocols.io.14egn2npyg5d/v2). Purified proteins were stored in GF buffer (10 mM HEPES, pH 7.4, 10% [v/v] glycerol, 150 mM NaCl, 1 mM TCEP) at −80 °C. Enzyme assays were performed in triplicates as described^7^. Briefly, 125 μL 0.2 M sodium phosphate buffer, pH 6.5, 15 μL enzyme and 10 µL *cis*-aconitate (neutralized, in water) were incubated at 37 °C for 10 min, followed by heat inactivation and HLPC quantification of itaconic acid. Assays were performed with seven different substrate concentrations. Curves of enzyme rate v over substrate concentration [S] were fitted using GraphPad Prism with the Michaelis–Menten equation v = *k*_*cat*_[S]/(*K*_*M*_ + [S]) to determine *k*_*cat*_ and *K*_*M*_.

### Transfections

Human epithelial A549 adenocarcinoma cells (DSMZ no. ACC107) were transfected using Lipofectamine LTX and PLUS Reagent (ThermoFisher #15338100) for 24 or 48 h and itaconate production was measured as described^8^ according to our validated LC–MS/MS assay^31^.

### Western blots

After aspirating the supernatant, the transfected A549 cells were washed once in ice-cold PBS and lysed in ice-cold RIPA buffer (containing protease inhibitor). Upon quantification of protein by Pierce BCA Protein Assay (ThermoFisher, #23225), lysates were diluted in 4× Laemmli sample buffer, then heat-denatured at 95 °C for 10 min. Equal amounts of protein extracts and 5 µl marker (PageRuler Plus Prestained Protein Ladder, #26620, ThermoFisher) were separated by 10% SDS-PAGE and transferred to a 0.45 µm nitrocellulose membrane (Amersham Protran Premium 0.45 µm NC Nitrocellulose Blotting Membrane, GE Healthcare #10600013). The membrane was blocked with 5% non-fat dry milk for 1 h at room temperature. After 3 × 10 min TBST (0.1% Tween 20 in TBS) washing, the membrane were incubated over night at 4 °C with primary antibodies specific to human ACOD1 (D6H2Y, Cell Signalling #77510; diluted 1:1000 in TBST), DYKDDDDK flag-tag (D6W5B, Cell Signaling, #14793S, diluted 1:1000 in 5% BSA), or Myc-tag (71D10, Cell Signalling #2278, diluted 1:1000 in 5% BSA in TBST), followed by incubation with the second antibody, goat anti-rabbit IgG-HRP (Southern Biotech #4030-05, diluted 1:5000 in TBST) for 1 h at room temperature. The bands were visualized by enhanced chemiluminescence via Amersham ECL Prime Western Blotting Detection Reagent (GE Healthcare #RPN2232). After stripping the membrane for 30 min at 60 °C in 62.5 mM Tris–HCl, 2% SDS, pH 6.8, β-actin bands were visualized using HRP-conjugated anti-β-Actin antibody (Abcam #ab49900, diluted 1:20,000 in TBST for 30 min at room temperature) via the ECL reagent.

### Neandertal and Denisovan ACOD1 sequence analysis

The ACOD1 coding sequences of Neandertals and Denisovans were analysed with the UCSC Genome Browser (https://genome.ucsc.edu) and with support by Janet Kelso (Max Planck Institute for Evolutionary Anthropology, Leipzig, Germany). The codon of Met154 was covered by one Neandertal sequence read and more than 20 reads for Denisovans.

## Supplementary Information

Supplementary Information.

## Data Availability

The raw data and materials that support the findings of this study are available from the corresponding authors upon reasonable request.
