# Supplementary material for: Amino acid positions near the active site determine the reduced activity of human ACOD1 compared to murine ACOD1
Source: Sci Rep. 2023 Jun 26;13:10360. doi: 10.1038/s41598-023-37373-w (PMC10293213; doi:10.1038/s41598-023-37373-w)
Supplement: Supplementary file 1 — Supplementary Information. [file 41598_2023_37373_MOESM1_ESM.pdf]

Supplementary Information

Amino acid positions near the active site determine the reduced activity of human ACOD1 compared to murine ACOD1

Fangfang Chen, Israfil Yalcin, Mingming Zhao, Chutao Chen, Wulf Blankenfeldt, Frank Pessler, Konrad Büssow

Supplementary Table 1. Data and statistical analysis for Figure 3a-d

	k_{cat} (95% CI) (s^{-1})	K_M (95% CI) (mM)	k_{cat}/K_M (95% CI) (s^{-1} M^{-1})	Itaconate (95% CI) (pmol μg^{-1})
**hACOD1**	0.83 (0.73-0.94)	0.45 (0.39-0.51)	1859 (1661-2057)	0.58 (0.51-0.65)
**mACOD1**	4.93 (3.81-6.05)**	0.65 (0.42-0.88)*	7611 (6468-8755)**	7.32 (7.09-7.55)****
**gACOD1**	4.41 (3.72-5.11)**	0.30 (0.19-0.41)**	14867 (10994-18739)**	1.68 (1.45-1.90)***
**Asn152Lys**	1.75 (1.64-1.87)****	0.30 (0.29-0.31)**	5771 (5251-6292)****	0.31 (0.26-0.36)***
**Met154Ile**	3.03 (2.51-3.55)**	0.34 (0.20-0.48)*	9072 (6874-11270)**	0.82 (0.80-0.85)**
**Met199Ile**	1.27 (0.98-1.56)**	0.65 (0.46-0.83)*	1988 (1052-2924)ns	0.16 (0.13-0.20)***
**Arg273Ser**	1.26 (1.13-1.39)****	0.25 (0.18-0.33)***	5031 (3264-6797)*	0.58 (0.51-0.65)ns
**Ser279Ala**	1.06 (0.96-1.15)**	0.29 (0.25-0.34)***	3638 (3242-4033)****	0.54 (0.45-0.64)ns
**Asn152Lys, Met154Ile**	4.58 (3.65-5.52)**	0.25 (0.16-0.33)***	18756 (14462-23049)**	0.91 (0.81-1.01)***

CI	confidence interval
P	P-value for comparison with hACOD1 values calculated with Welch's unequal variances t-test
ns	$P > 0.05$
*	$P \leq 0.05$
**	$P \leq 0.01$
***	$P \leq 0.001$
****	$P \leq 0.0001$

Supplementary Table 2. MetAromatic output for 6R6U

ARO	POS	MET POS	NORM	MET-THETA	MET-PHI
PHE	134	33	4.675	58.534	156.413
PHE	381	154	4.596	92.552	73.826
PHE	381	154	3.94	87.844	63.705
PHE	381	154	3.65	100.532	47.894
PHE	381	154	4.094	116.647	49.048
PHE	381	154	4.739	117.581	62.157

Output of the MetAromatic program for the PDB structure 6R6U with default cutoff values. ARO, aromatic residue, POS, aromatic residue number, MET POS, methionine position. The NORM column specifies the distance (in Å) between the Met residue and one of the midpoints between two carbon atoms in an aromatic ring. MET-THETA and MET-PHI are the angles of the sulfur's free electron pairs with that midpoint.

Supplementary Table 3. Plasmids

Plasmid	Plasmid name	Open Reading Frame	QuikChange Primers	Reference
**pCAD29**	pCAD29_hIRG1_4-461_pvp008	hACOD1 aa 4-461, N-term. StrepTagII and TEV		[1]
**pCAD39**	pCAD39_mIRG1_4-462_pvp008	hACOD1 aa 4-462, N-term. StrepTagII and TEV		[1]
**pCMV6Entry-hIrg1**	pCMV6Entry-hIrg1	full length hACOD1, C-term. Myc-tag, Flag-tag		[1]
**pCMV6Entry-mIrg1**	pCMV6Entry-mIrg1	full length mACOD1, C-term. Myc-tag, Flag-tag		[1]
**pCAD130**	pCAD130_hIrg1_M154I_pvp008	same as pCAD29	hCADzuMaus_Met_S/AS	This study
**pCAD122**	pCAD122_hIrg1_N152K_pvp008	same as pCAD29	hCADzuMaus_t456g_S/AS	This study
**pCAD136**	pCAD136_hIrg1_M199I_pvp008	same as pCAD29	hCADm2_M199I_g597a_S/AS	This study
**pCAD121**	pCAD121_hIrg1_R273S_pvp008	same as pCAD29	hCADzuMaus_c817a_S/AS	This study
**pCAD134**	pCAD134_hIrg1_S279A_pvp008	same as pCAD29	hCADm2_S/AS279A_t835g_S/AS	This study
**pCAD123**	pCAD123_hIrg1_N152K,M154I_pvp008	same as pCAD29	hCADzuMaus_2mut2_S/AS	This study
**pCAD183 (gACOD1)**	pCAD183_gCAD_pET	gACOD1, aa 4-461, N-term. StrepTagII and TEV		This study
**pCAD161 (cACOD1)**	pCAD161_cCAD_pET	cACOD1 aa 12-469, N-term. StrepTagII and TEV		This study
**pCAD131**	pCAD131_hIrg1_M154I_pCMV6	pCMV6Entry-hIrg1	hCADzuMaus_Met_S/AS	This study
**pCAD126**	pCAD126_hIrg1_N152K_pCMV6	pCMV6Entry-hIrg1	hCADzuMaus_t456g_S/AS	This study
**pCAD137**	pCAD137_hIrg1_M199I_pCMV6	pCMV6Entry-hIrg1	hCADm2_M199I_g597a_S/AS	This study
**pCAD125**	pCAD125_hIrg1_R273S_pCMV6	pCMV6Entry-hIrg1	hCADzuMaus_c817a_S/AS	This study
**pCAD135**	pCAD135_hIrg1_S279A_pCMV6	pCMV6Entry-hIrg1	hCADm2_S/AS279A_t835g_S/AS	This study
**pCAD127**	pCAD127_hIrg1_N152K,M154I_pCMV6	pCMV6Entry-hIrg1	hCADzuMaus_2mut2_S/AS	This study
**pCAD182 (gACOD1)**	pCAD182_gCAD_pCMV6-entry	pCMV6Entry-hIrg1		This study
**pCAD180 (cACOD1 11-490)**	pCAD180_cCAD_11-end_pCMV6	pCMV6Entry-hIrg1		This study
**pCAD181 (cACOD1 1-490)**	pCAD181_cCAD_1-end_pCMV6	pCMV6Entry-hIrg1		This study

Supplementary Table 4, QuikChange Primers

Primer Name	Mutation	Sequence
**hCADzuMaus_t456g_S**	N152K	GGAGGCCAAGGACATGCCAAAGAGATTCC
**hCADzuMaus_t456g_AS**	N152K	TGGCATGTCCTTGGCCTCCTTGGCGAAATG
**hCADzuMaus_Met_S**	M154I	GGAGGCCAATGACATACCAAAGAGATTCCATCCCCCTTC
**hCADzuMaus_Met_AS**	M154I	GAATCTCTTTGGTATGTCATTGGCCTCCTTGGCGAAATG
**hCADm2_M199I_g597a_S**	M199I	GGGGCACCCATAGCCAATGCTGCCACCC
**hCADm2_M199I_g597a_AS**	M199I	ATTGGCTATGGGTGCCCCAGCATGGGAAAC
**hCADzuMaus_c817a_S**	R273S	CGTGGCCTTTAAGAGTTTTCCTGCACATTTATCTACC
**hCADzuMaus_c817a_AS**	R273S	GTGCAGGAAAACTCTTAAAGGCCACGTCCTGCTGG
**hCADm2_S279A_t835g_S**	S279A	TTTTCCTGCACATTTAGCTACCCACTGGGTGGCAG
**hCADm2_S279A_t835g_AS**	S279A	GTGGGTAGCTAAATGTGCAGGAAAACGCTTAAAGGCC
**hCADzuMaus_2mut2_S**	N152K,M154I	GGAGGCCAAGGACATACCAAAGAGATTCCATCCCCCTTC
**hCADzuMaus_2mut2_AS**	N152K,M154I	GAATCTCTTTGGTATGTCCTTGGCCTCCTTGGCGAAATG

Supplementary Table 5. PCR Primers

Primer Name	Primer sequence
**cCAD-For1-GoldMut**	GCGGGTACCGGTCTCAAATGGCTAGCTGGAGCCACCCGC
**Gold3-cCAD-For2**	GCGGGTACCGGTCTCACAAGATCTACAGTTCCAACATATCCAGCACTGTTTGGGGTCGCCCAGACATCAGGCTCCCGCCC
**Gold3-cCAD-For3**	GCGGGTACCGGTCTCAACGCGCCACCCTGCCACCCACCCTTCTG
**Gold3-cCAD-For4**	GCGGGTACCGGTCTCAGATTTGGGGCCTTTTATGCCAACTATTCCCCAAAAGTCCTTCCAAGCCTGGCTTCCTACAGTTGGCTGCTGGA
**Gold3-cCAD-For5**	GCGGGTACCGGTCTCACAATTGACTACATTAAGAGAATTGTGCTCAGG
**Gold3-cCAD-For6**	GCGGGTACCGGTCTCACACCCTGTACTGTGAAATAAGTGTCACCCTC
**Gold3-cCAD-Rev1**	GCGGGTACCGGTCTCTCTTGCTATATTGGCTGGCTATGTGAAACACTTCCATAGTGGTTCCCAGGAACCCAGCACC
**Gold3-cCAD-Rev2**	GCGGGTACCGGTCTCTGCGTGTCATCAAAATCCATGGAGTGAATAGCCA
**Gold3-cCAD-Rev3**	GCGGGTACCGGTCTCTAATCCTGCCTCCAAGTCCAAGACCTGCTTGTTTCCTTGCAGACCCAACATTGCCAAAAATGCAGCTT
**Gold3-cCAD-Rev4**	GCGGGTACCGGTCTCTATTGGAAGCAGGGCTCTCTCTGCTA
**Gold3-cCAD-Rev5**	GCGGGTACCGGTCTCTGGTGTTGAAGCTTGGCAAGTTGTCCG
**Gold3-cCAD-Rev6**	GCGGGTACCGGTCTCTACCTTCAGGGTCCTTTGAGAAGTGTAGTTAACACAGAACAGTCTTCTAGGTCTTCTAGATTTTCGACTATCTTTATAAGGCTTTCCACTGTG
**cCAD-pCMV6-LF**	CTATAGGGCGGCCGGGAATTCATGATGTACCCTGTCCTAACACTCTTCCCTCTCAAGTCTATCACAGAAAGCTTTGCC
**cCAD-pCMV6-SF**	CTATAGGGCGGCCGGGAATTCATGATGCTCAAGTCTATCACAGAAAGCTTTGCC
**cCAD_1-480_pCMV6_R**	GAGTTTCTGCTCGAGCGGCCGCTCTGGAGAGTTTGAAGCTACCTCTGGTGGAGAGGGTCCTTTGAGAAGTGTAGT
**cCAD_pCMV6_fl_R**	GAGTTTCTGCTCGAGCGGCCGCTTTGCTGCTGTTCTTATGTTAGTGAGTGCGTTCTCTGGAGAGTTTGAAGCTACCT
**gCAD_CAD177_VLTILLK_R**	CACCACAGGTCTCGACCTTCAGGGTCCTTTGAGAAGTATAGTTAACACAGAACAGTCTTCTAGGT
**gCAD_pCMV6_F1_CAD177**	CGACTCACTATAGGGCGGCCGGGAATTCATGATGCTCAAGTCTATCACAGAAAGCTTTGCC
**gCAD_pCMV6_F2_hIrg1**	TGTTAACTATACTTCTCAAAGGACCCTCTCCAC
**gCAD_pCMV6_R1_CAD177**	CCTTTGAGAAGTATAGTTAACACAGAACAGTCTTCTAGGT
**gCAD_pCMV6_R2_hIrg1**	GTTTCTGCTCGAGCGGCCGCTTGGAGAGATTTGTGATAGAA

Supplementary Table 6. PCRs for cloning

PCRs for cloning of pCAD161 (cACOD1 in pET-T7pro-ter)

F primer	R primer	template	cloning method
**cCAD-For1-GoldMut**	Gold3-cCAD-Rev1	pCAD130	Golden Mutagenesis
**Gold3-cCAD-For2**	Gold3-cCAD-Rev2	pCAD130	Golden Mutagenesis
**Gold3-cCAD-For3**	Gold3-cCAD-Rev3	pCAD130	Golden Mutagenesis
**Gold3-cCAD-For4**	Gold3-cCAD-Rev4	pCAD130	Golden Mutagenesis
**Gold3-cCAD-For5**	Gold3-cCAD-Rev5	pCAD130	Golden Mutagenesis
**Gold3-cCAD-For6**	Gold3-cCAD-Rev6	pCAD130	Golden Mutagenesis

PCRs for cloning of pCAD177 (pET-T7pro-ter)

F primer	R primer	template	cloning method
**cCAD-For1-GoldMut**	Gold3-cCAD-Rev3	pCAD130	Golden Mutagenesis
**Gold3-cCAD-For4**	Gold3-cCAD-Rev5	pCAD130	Golden Mutagenesis
**Gold3-cCAD-For6**	Gold3-cCAD-Rev6	pCAD130	Golden Mutagenesis

PCRs for cloning of pCAD183 (gACOD1 in pET-T7pro-ter)

F primer	R primer	template	cloning method
**cCAD-For1-GoldMut**	gCAD_CAD177_VLTILLK_R	pCAD177	Golden Gate

PCRs for cloning TOPO4

F primer	R primer	template	cloning method
**cCAD-pCMV6-LF**	cCAD_1-480_pCMV6_R	pCAD161	Zero Blunt TOPO cloning

PCRs for cloning TOPO5

F primer	R primer	template	cloning method
**cCAD-pCMV6-SF**	cCAD_1-480_pCMV6_R	pCAD161	Zero Blunt TOPO cloning

PCR for cloning pCAD181 (cACOD1.1 in pCMV6Entry)

F primer	R primer	template	cloning method
**cCAD-pCMV6-LF**	cCAD_pCMV6_fl_R	TOPO4	SLIC

**PCR for cloning pCAD180 (cACOD1.11 in pCMV6Entry)**			
**F primer**	**R primer**	**template**	**cloning method**
**cCAD-pCMV6-SF**	cCAD_pCMV6_fl_R	TOPO5	SLIC

**PCR for cloning pCAD182 (gACOD1 in pCMV6Entry)**			
**F primer**	**R primer**	**template**	**cloning method**
**gCAD_pCMV6_F1_CAD177**	gCAD_pCMV6_R1_CAD177	CAD177	SLIC
**gCAD_pCMV6_F2_hIrg1**	gCAD_pCMV6_R2_hIrg1	hIrg1-pCMV6-entry	SLIC

Supplementary Figure 1

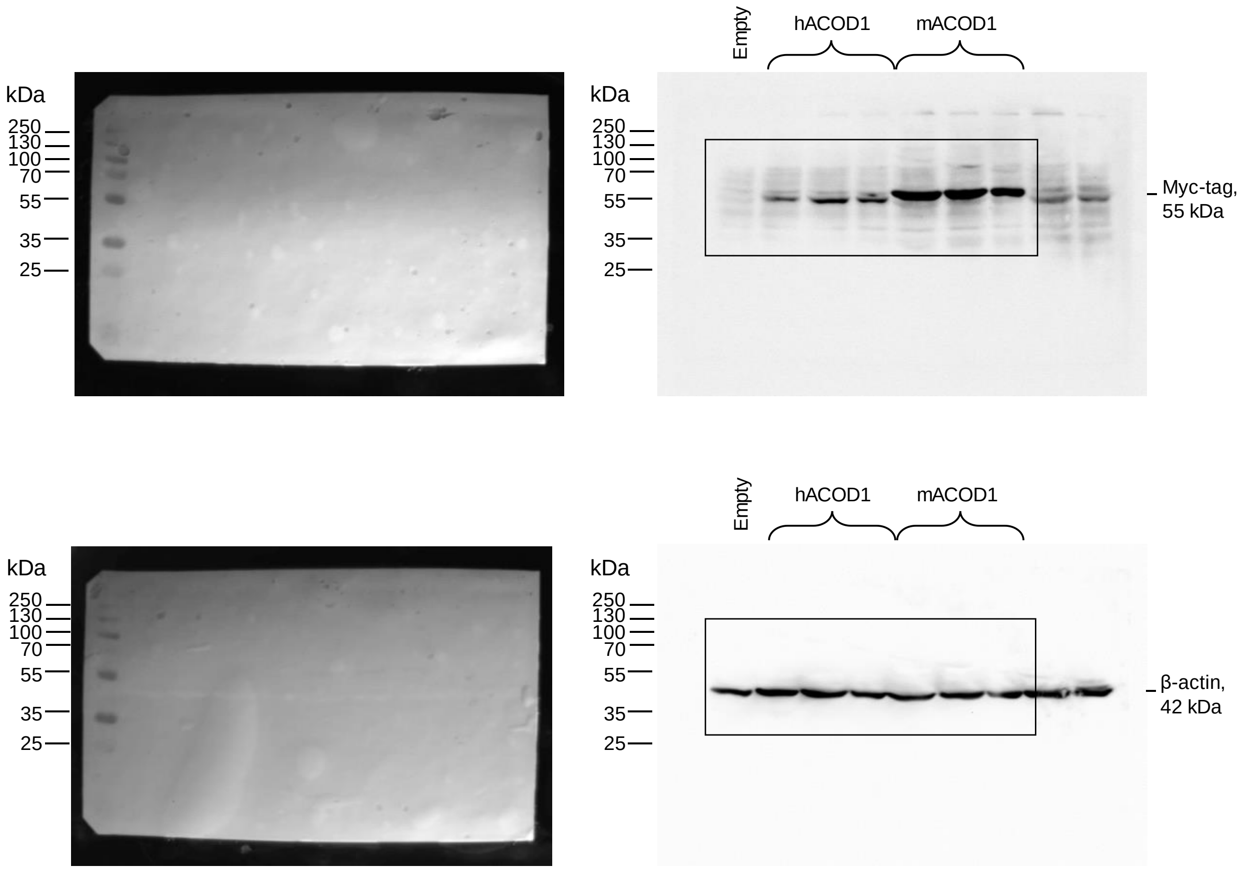

Original blots for Figure 3e. Cropped regions are indicated by boxes. Uncropped membranes with visible marker bands (left) and corresponding chemiluminenscence images (right) are shown.

Supplementary Figure 2

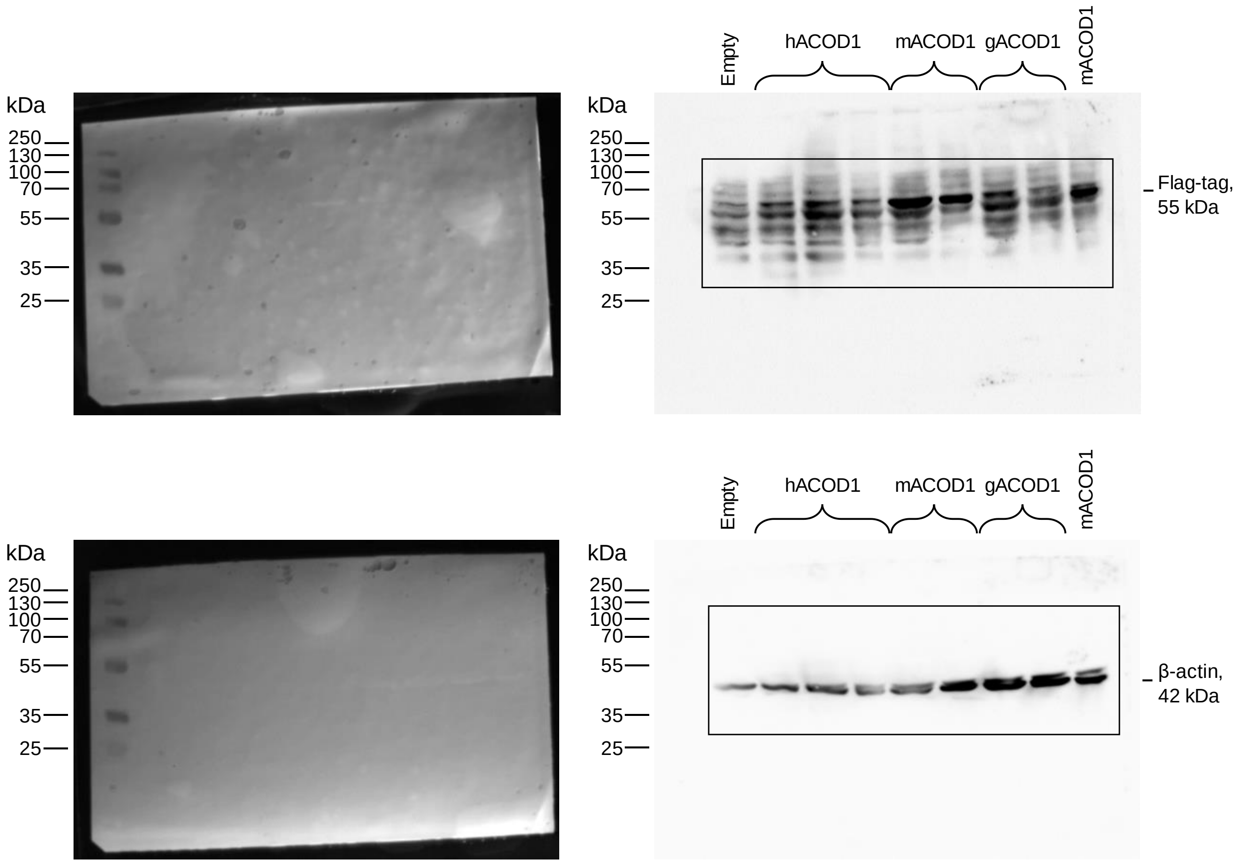

Original blots for Figure 3f. Cropped regions are indicated by boxes. Uncropped membranes with visible marker bands (left) and corresponding chemiluminenscence images (right) are shown.

Supplementary Figure 3

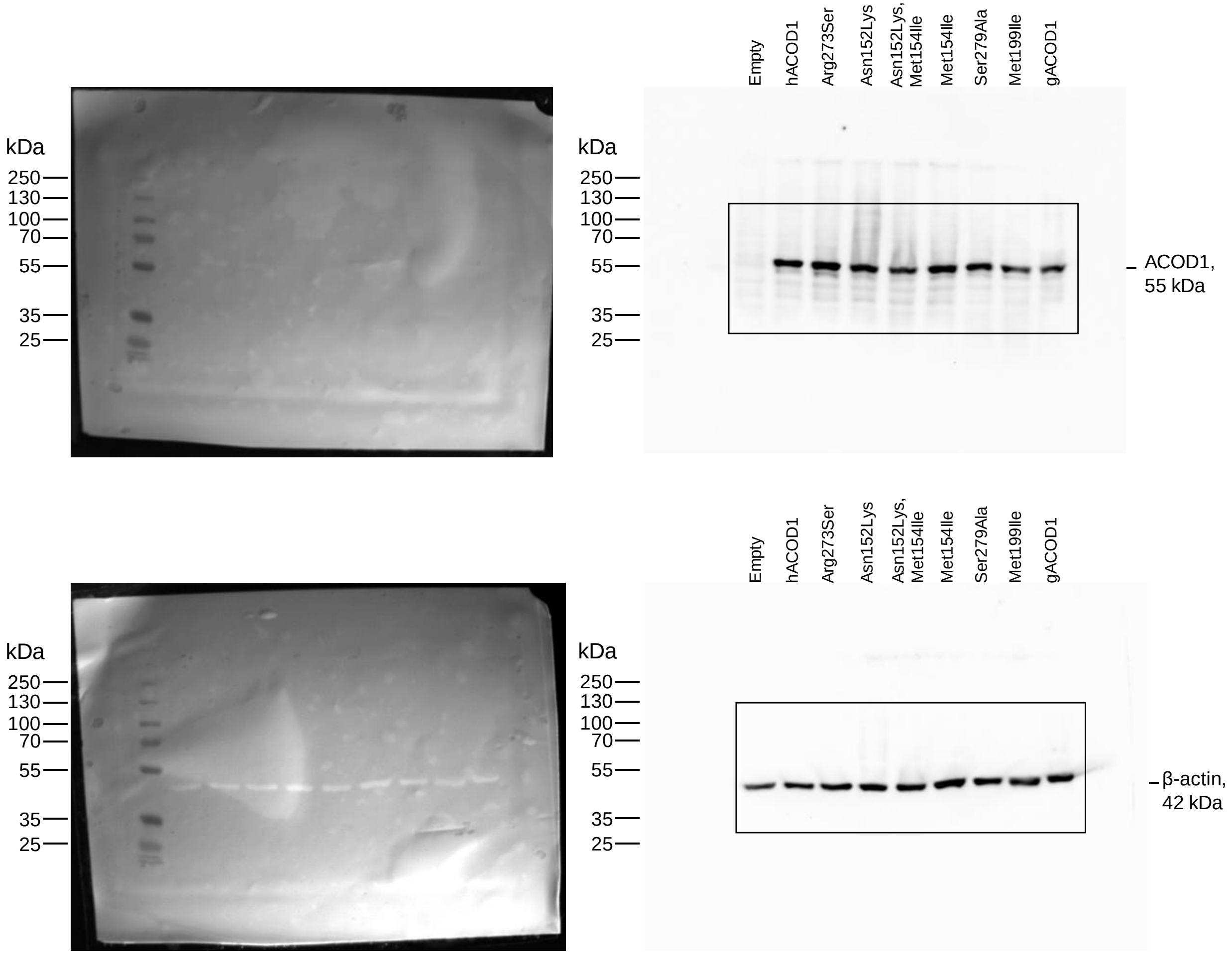

Original blots for Figure 3g. Cropped regions are indicated by boxes. Uncropped membranes with visible marker bands (left) and corresponding chemiluminenscence images (right) are shown.

References

1. Chen, F., *et al.* Crystal structure of cis-aconitate decarboxylase reveals the impact of naturally occurring human mutations on itaconate synthesis. *Proc. Natl. Acad. Sci. U. S. A.* **116**, 20644-20654 (2019).
